# Supplementary material for: Development of an artificial intelligence system to identify hypoglycaemia via ECG in adults with type 1 diabetes: protocol for data collection under controlled and free-living conditions
Source: BMJ Open. 2023 Apr 18;13(4):e067899. doi: 10.1136/bmjopen-2022-067899 (PMC10124264; doi:10.1136/bmjopen-2022-067899)
Supplement: Author's manuscript [file bmjopen-2022-067899.draft_revisions.pdf]

BMJ Open is committed to open peer review. As part of this commitment we make the peer review history of every article we publish publicly available.

When an article is published we post the peer reviewers' comments and the authors' responses online. We also post the versions of the paper that were used during peer review. These are the versions that the peer review comments apply to.

The versions of the paper that follow are the versions that were submitted during the peer review process. They are not the versions of record or the final published versions. They should not be cited or distributed as the published version of this manuscript.

BMJ Open is an open access journal and the full, final, typeset and author-corrected version of record of the manuscript is available on our site with no access controls, subscription charges or pay-per-view fees (http://bmjopen.bmj.com).

If you have any questions on BMJ Open's open peer review process please email info.bmjopen@bmj.com

BMJ Open

Prospective data collection for the development and validation of artificial intelligence models for non-invasive glycaemic event detection using ECG in people with type 1 diabetes: study protocol

Journal:	*BMJ Open*
Manuscript ID	bmjopen-2022-067899
Article Type:	Protocol
Date Submitted by the Author:	05-Sep-2022
Complete List of Authors:	Cisuelo, Owain; University of Warwick, School of Engineering<br>Stokes, Katherine; University of Warwick,<br>Oronti, Busola; University of Warwick, School of Engineering<br>Haleem, Salman; University of Warwick, Advanced Biomedical Signal Processing and Intelligent eHealth Lab<br>Barber, Thomas; University Hospitals Coventry and Warwickshire NHS Trust; University of Warwick<br>Weickert, Martin; University Hospitals Coventry & Warwickshire NHS Trust, ; University of Warwick Warwick Medical School, Division of Translational and Systems Medicine<br>Pecchia, Leandro ; University of Warwick, School of Engineering<br>Hattersley, John; University Hospitals Coventry & Warwickshire NHS Trust, Human Metabolism Research Unit; University of Warwick, School of Engineering
Keywords:	Health informatics < BIOTECHNOLOGY & BIOINFORMATICS, General diabetes < DIABETES & ENDOCRINOLOGY, General endocrinology < DIABETES & ENDOCRINOLOGY

SCHOLARONE™
Manuscripts

Title	Prospective data collection for the development and validation of artificial intelligence models for non-invasive glycaemic event detection using ECG in people with type 1 diabetes: study protocol
Authors' names	Owain Cisuelo[1*], Katy Stokes[1], Busola Oronti[1], Muhammad Salman Haleem[1], Thomas M Barber[2,3,4], Martin O Weickert[2,3,4], Leandro Pecchia[1], John Hattersley[1,4]
Authors' address	1. School of Engineering, University of Warwick, Coventry, CV4 7AL, UK<br>2. Medical School, University of Warwick, Coventry, CV4 7HL, UK<br>3. Department of Endocrinology and Diabetes, University Hospitals Coventry & Warwickshire NHS Trust, Coventry, CV2 2DX, UK<br>4. Human Metabolism Research Unit, University Hospitals Coventry & Warwickshire NHS Trust, Coventry, CV2 2DX, UK
Corresponding author	*Correspondence to Owain.Cisuelo@Warwick.ac.uk
Word count	2,750

For peer review only

Abstract

Introduction Hypoglycaemia is a harmful potential complication in people with Type 1 diabetes mellitus (T1DM) and can be exacerbated in patients receiving treatment, such as insulin therapies, by the very interventions aiming to achieve optimal blood glucose levels. Symptoms can vary greatly, including, but not limited to, trembling, palpitations, sweating, dry mouth, confusion, seizures, coma, brain damage or even death if untreated. A pilot study with healthy (euglycaemic) participants demonstrated that hypoglycaemia can be detected non-invasively with Artificial Intelligence (AI) using physiological signals obtained from wearable sensors. This protocol provides a methodological description of an observational study in which we aim to obtain physiological data from people with T1DM to validate and improve the AI for use in this population. We also aim to investigate a general AI model for glycaemic event detection. Such a model could be suitable for integrating into a continuous, non-invasive, glucose monitoring system, contributing towards improving surveillance and management of blood glucose for people with diabetes.

Methods and analysis This observational study aims to recruit thirty T1DM patients from a diabetes outpatient clinic at the University Hospital Coventry and Warwickshire for a two-phase study. The first phase involves attending an inpatient protocol for up to thirty-six hours in a calorimetry room under controlled conditions, followed by a phase of free-living, for up to three days, in which participants will go about their normal daily activities unrestricted. Throughout the study the participants will wear wearable sensors to measure and record physiological signals (e.g., electrocardiogram and continuous glucose monitor). Data collected will be used to develop and validate an AI model using state-of-the-art deep-learning methods.

Ethics and dissemination This study has received ethical approval from National Research Ethics Service (Ref: 17/NW/0277). The findings will be disseminated via peer-reviewed journals and presented at scientific conferences.

Trial registration number NCT05461144

Keywords Artificial Intelligence (AI); Deep learning; Non-invasive glycaemic event detection; Hypoglycaemia detection; ECG signal processing; Type 1 diabetes mellitus (T1DM).

Article Summary

Strengths and limitations of this study

- We aim investigate the performance of an artificial intelligence system for automatic non-invasive glycaemic event detection using continuous physiological data obtained from people with type 1 diabetes in real-life settings.
- Using quantitative analysis, we will investigate the correlative relationship between parameters of the electrical activity of the heart and changes in glucose concentration.
- This is a single centre observational study aiming to recruit from the local population without discrimination of characteristics such as sex, ethnicity or race which may limit the generalisability of the results.
- We will use a wearable continuous glucose monitoring sensor to measure glucose in the interstitial fluid which has a physiologic time delay compared to blood glucose.

Introduction

Type 1 Diabetes Mellitus (T1DM) is a chronic disorder caused by autoimmune damage to the insulin producing pancreatic beta cells, leading to elevated blood glucose concentration (hyperglycaemia)[1]. T1DM requires exogenous insulin treatment[2], of which hypoglycaemia is a potential side effect[3]. The prevalence of T1DM globally is 5.9 per 10,000 with the incidence of 15 per 100,000 people[4 5]. In UK, the prevalence is reported as 5th highest with 8.6% and incidence of T1DM is rising 5% every year[4]. There is no cure for diabetes, therefore, effective management can be pivotal in reducing risk of adverse events as well as delay the onset of long-term complications [6 7].

The self-monitoring of blood glucose (SMBG) can play a key role in effective management of diabetes. SMBG can empower patients to adhere to lifestyle interventions, such as changes to diet and exercise regimen, as they have access to immediate feedback on the effects[8]. Active participation from the patient in their care has been

For peer review only - http://bmjopen.bmj.com/site/about/guidelines.xhtml

shown to improve outcomes such as reducing the risk of diabetic complications[9]. Current methods for glucose monitoring have several limitations. The most common method for SMBG is an invasive 'finger prick test' in which a small sample of blood is collected from the fingertip and analysed by a handheld device called a glucometer. This invasive procedure has several documented barriers preventing SMBG such as inconvenience, cost, and discomfort[10]. Moreover, the devices and associated consumables are prone to error if not operated correctly[11], requiring repeat attempts and generating unnecessary waste. Hypoglycaemia typically occurs during sleep[12], and those receiving insulin-therapy are at increased risk due to a combination of factors such as impaired counterregulatory-hormone response and warning symptoms going unobserved[13]. This presents an obvious limitation to the effectiveness of an invasive 'finger prick test' as a tool for SMBG. An alternative to 'finger pricking' are continuous glucose monitoring (CGM) devices which require a small cannula inserted in the top layer of skin to continuously measure glucose in the fluid between the cells. They can be described as minimally invasive as after the initial fitting they are routinely worn continuously for up to fourteen days. However, despite being shown to be a beneficial tool for SMBG, national clinical guidelines for the UK do not recommend routine use of CGMs for the management of T1DM due to insufficient evidence demonstrating their efficacy and cost-effectiveness[14].

It has been demonstrated, by this group[15 16] and others[17-19], that glycaemic events can affect certain cardiac characteristics, which can be extracted from readings of the electrical activity of the heart, the electrocardiogram (ECG). With increasing availability of wearable devices for tracking physiological signals, studies have been undertaken to investigate prediction of blood glucose concentration or glycaemic event detection using artificial intelligence (AI), by combining data or features extracted from the ECG with blood glucose measurements. Such an approach would address the need for non-invasive continuous blood glucose monitoring. Studies have shown promising predictive performance based on data collected from healthy subjects[15 16 20-22], or adolescent T1DM patients[19 23]. In a review of techniques for detecting hypoglycaemia, Diouri et. al, note that the use of ECG-based techniques has only been investigated in small cohort trails, and that the success of such approaches relies on further, ideally larger, trials and validation in patients with diabetes and cardiac diseases[24].

This protocol expands on an initial pilot, results of which are published[15], which made opportune use of applicable secondary data collected for a separate study[25] on healthy elderly males. The aim of the pilot was to use the data to create a personalised AI model for the detection of nocturnal hypoglycaemia. The developed and validated deep-learning model achieved 90% for specificity (ability to identify true positives) and sensitivity (ability to identify true negatives) which are clinically relevant metrics in relation to diagnosis of disease[26]. The aim of this protocol is to build on this initial work, by obtaining new data to validate the technique for people with diabetes and test its applicability beyond controlled conditions, to that of normal living conditions.

Study Objective

The purpose of this two-phase observational study is to develop and validate our novel AI models for non-invasive detection of glycaemic events, such as hypoglycaemia, using physiological signals and to infer physiological responses to physical activities and diet. To achieve this, we will monitor glucose fluctuations and ECG variability in people with T1DM who will also be asked to record diaries to track lifestyle activities. The physiological signals will be recorded via non-invasive wearable devices and glycaemic events will be based on glucose measured in the interstitial fluid monitored via CGM.

Methods and analysis

Study setting

This two-phase observational study will be conducted at the Human Metabolism Research Unit (HMRU) and in free-living conditions. During phase 1, the participants attend an inpatient protocol at University Hospital Coventry and Warwickshire (UHCW) in a calorimetry room for up to thirty-six hours. The calorimetry room is a controlled environment which allows for precise regulation of parameters such as ambient temperature, air pressure, humidity, and subject behaviour enabling us to obtain high quality baseline ECG and glucose data from the wearable sensors. In phase 2 the same sensors will be used to collect data under free-living conditions, during

For peer review only

which the participants are free to go about their normal daily activities without restriction. Participants will be asked to complete diaries for daily activity, food intake and sleep.

Inclusion and Exclusion Criteria

The study will be open to all adult individuals living independently with T1DM who are comfortable with a stay in a calorimetry room. The inclusion and exclusion criteria are summarised in table 1.

Table 1. Inclusion and Exclusion Criteria

**Inclusion Criteria**
• Aged 18 years or older<br>• Without acute illness or ongoing clinical investigation<br>• Participants with an ongoing medical condition will only be included after detailed consultation with clinical and dietetics members of the team
**Exclusion Criteria**
• Children (under 18 years)<br>• Any adult who lacks decisional capacity<br>• Claustrophobic and/or isolophobic patients, or needle phobia<br>• Individuals who have undertaken recent abnormal exercise, radiation exposure within the preceding 24 hours of entering the whole-body calorimeter and feeling unwell in any way.<br>• Any medical/endocrine problem that could affect energy expenditure (e.g., thyroid problems, Cushing's syndrome)<br>• Chronic inflammatory disorders like rheumatoid arthritis, or long-term use of steroids or other immunomodulators like cyclosporine, azathioprine.<br>• Beta blockers<br>• Currently actively losing weight<br>• Depression or any psychiatric illness

Enrolment procedure

Participants will be recruited from the Warwickshire Institute for the Study of Diabetes, Endocrinology, and Metabolism clinic at UHCW. We aim to recruit up to thirty adult T1DM patients. It is anticipated that recruitment will be an ongoing process and we will begin to identify potential participants from September 2022. All participants will receive a participant information sheet and written informed consent will be obtained

Sample size

As this is an observational study, a sample size calculation is not possible. A target of thirty participants will be enrolled, based on a pragmatic approach which ensures sufficient data is available for analysis.

Study Protocol

The study setting, protocol, and wearing of devices are shown in figure 1.

Calorimetry room

The participant will stay in the calorimetry room for up to thirty-six hours, during which there will be set times for meals, light exercise, and rest. Meals will be provided with known total energy and macronutrient content. Water will be provided ab libitum. While in the calorimetry room, wearable sensors will record physiological data continuously.

Other activities will be performed during this time, specifically: a) Up to 12 serial blood samples of 30ml will be obtained from a peripheral venous catheter inserted at the beginning of phase 1 to measure venous glucose and insulin concentration, b) finger-prick blood samples will be taken periodically for capillary glucose measurements, c) up to 12 saliva samples will be taken to analyse salivary concentration of cortisol and melatonin, d) all urine will be collected as voided for protein oxidation analysis, e) movement and activity will be assessed by motion sensors, f) simple questionnaires will be used to measure food intake and activity as well as subjective

For peer review only

aspects such as appetite, satiety, and wellness, and g) daytime blood pressure will be monitored using an ambulatory blood pressure monitor (hourly readings).

Free-Living

While free-living, the participant will continue to wear the monitoring devices to record ECG and glucose concentration. If tolerated, participants will also be fitted with an ambulatory blood pressure monitor for the first 24-hours of the free-living phase. Instructions will be provided on how to affix and operate the devices. During this time, participants will also be required to keep brief diaries detailing physical activity, food intake and sleep. The first is a daily activity diary, to be completed at the end of each day, detailing: any device removal and replacement, physical activity, food intake, alcohol intake and caffeine intake. The second is a standardised sleep diary, the Consensus Sleep Diary-M[27] to be completed on waking in the morning.

Figure 1. During phase 1 the participant is fitted with and trained in the use of the wearable devices. At the HMRU they will partake in several other activities such as light exercise and eating of preprepared meals. Phase 1 is followed by phase 2 where the participant will continue to wear the sensors while going about their normal daily activities for up to 3 days.

Devices

Continuous Glucose Monitoring

Continuous glucose levels will be measured using a FreeStyle Libre 2 flash glucose monitoring system, which can be worn for up to fourteen days. Although the sensor is sampling continuously, the glucose concentration is reported in fifteen-minute intervals. The sensor can store data for a period of eight hours. As such, data will be extracted at regular intervals using a smartphone preconfigured with the companion application. The sensor is water-resistant and can be used while bathing, showering, swimming, or exercising, and is worn on the back of upper arm. The device has been evaluated for accuracy and user experience and found to have been generally well received by T1DM patients who used it for ten to fourteen days [28].

ECG

Medtronic Zephyr BioPatch is a CE marked device, indicating that the product complies with EU safety, health, and environmental requirements, which operates across one lead within an ECG amplitude range between 0.25 and 15 mV, with a sampling frequency of 250Hz. The device will be affixed to the wearer's skin via two electrodes placed in the centre of the chest. It can also be worn in a secondary configuration attached to a fabric harness which is worn across the chest if the electrodes are not tolerated. The device will be removed before bathing, showering, or swimming and reaffixed afterwards. The battery is rated for thirty-five hours of continual use. Each participant will be provided with fully charged devices for the free-living phase of the study.

Data Management

All data collected is owned by the UHCW National Health Service (NHS) Trust. All electronic data, physiological or otherwise, generated as part of this study will be anonymised and stored in the HMRU database on secure servers and backed up and protected in accordance with NHS guidelines. Anonymised data will be made available to researchers for analysis under an Institutional Data Sharing Agreement between the UHCW and University of Warwick. Data will extracted from the devices, anonymised, stored, and made available for analysis when the participant has concluded the protocol.

Data Analysis and Modelling

Data Processing

For peer review only

The effect of glycaemic events on the ECG signals will be examined. It is anticipated that ECG signals will be affected by noise and artefacts due to body movement or heavy respiration. Therefore, we will apply pre-processing methods such as baseline wander removal to the raw signal to remove low frequency noise. We will then identify individual heart beats in the ECG and detect fiducial points using our ECG segmentation tool developed in a recent study for cardiovascular disease detection[29]. Heartbeats will then be grouped into fifteen-minute excerpts, corresponding to the sampling of the CGM, and annotated with the glycaemic state (i.e., hypoglycaemic, normal, hyperglycaemic)[15]. Due to the lower sample frequency of the CGM, we will perform linear interpolation to estimate glucose measurements at every second.

Data Analysis

Characteristics of an ECG can be represented by heart rate variability (HRV) parameters and can be categorised into time-domain features, frequency-domain features and non-linear features[30]. We aim to use physiological signal processing packages such as NeuroKit2[31] and HeartPy[32] to determine these features. Furthermore, we aim to determine ECG beat parameters which include length and slope among the fiducial points (P, Q, R, S and T). The fiducial points can be detected using our deep learning based ECG segmentation tool[29]. We will perform statistical tests to determine the statistical significance of different HRV features and ECG beat parameters among different glycaemic values to determine which parameters are associated with hypoglycaemia.

Data Modelling

The inherent part of the project is to develop the AI models for predicting glycaemic events and inferring them with physical activity and diet. The state-of-the-art AI models vary from traditional machine learning models (such as Support Vector Machines, Decision Trees etc.) to advanced deep learning based models[33]. Traditional machine learning models have the capability to train static features for the development of explanatory models whereas deep learning models can train raw ECG signals based on spatial and temporal context. The deep learning models will be built upon on earlier work developed for healthy subjects[15]. We will use well-documented frameworks and packages such as TensorFlow[34], Scikit-learn[35], and pandas[36].

Outcomes

Primary Outcome

The primary outcome of this study is to evaluate the performance of a robust predictive model for detecting glycaemic events non-invasively from the ECG signal for a T1DM cohort. We will extract and analyse HRV features and ECG beat parameters from the raw signal and determine their relationship with the glycaemic status. A subset of the data will be used to train the model by using state-of-the-art algorithms to create a mathematical mapping from the ECG-derived features to the glycaemic status at that time index. The remaining data can then be used to test the how the model performs on new data. The glycaemic status for the T1DM cohort will be detected non-invasively via ECG signals by the automated AI algorithm which will be trained using the glycaemic event thresholds defined in Table 2. In addition to validating the hypoglycaemia detection model for the T1DM cohort, we also aim to develop a general glycaemic event detection algorithm.

Table 2. Glycaemic event thresholds for people with T1DM[37-43].

Glycaemic event	Blood glucose concentration (mmol/L)
Severe hypoglycaemia	<2.8
Hypoglycaemia	>2.8 and <3.9
Euglycaemia	>3.9 and <11.1
Hyperglycaemia	>11.1 and <13.9
Severe hyperglycaemia	>13.9

Secondary Outcome

The secondary outcome of this study is to develop the statistical inferences of the impact of physical activity and diet on glycaemic events. We will evaluate glycaemic events as predicted by ECG beat parameters, HRV features and ECG signals which would be further correlated by food and physical activity diaries.

For peer review only

Patient and public involvement

No formal PPI group was convened for this study, however, phase 1 is a standard protocol used at the HMRU and has been continually developed with PPI for over 10 years. The methodology using a single-lead wearable ECG device and CGM was discussed and reviewed with participants for a previous study investigating the effects of resistance exercise and protein supplementation on sarcopenia in healthy older men (clinical trials identifier: NCT03299972). The PPI consists of informal, non-scripted interviews with members of the public post-study via the UHCW PPI Forum. Depending on the results obtained, PPI groups may be approached to discuss non-scientific dissemination.

Discussion

The primary objective of this observational study is to obtain physiological data from people with T1DM using non-invasive wearable sensors to validate and further develop an AI model for automated detection of hypoglycaemia. The initial model [15] was developed using data from healthy individuals and the results determined that the personalised classifiers based on deep-learning algorithms can reliably perform automatic detection of hypoglycaemic events using features extracted from the ECG waveform recorded with wearable devices. This study is designed to improve the performance and robustness of this AI model for use in a population with T1DM.

Additionally, as a secondary outcome, this study aims to develop a general algorithm for the detection of glycaemic events in both diabetic and healthy subjects using the data collected from this study and the pilot. The development of an accurate and robust model for the non-invasive detection of abnormal excursions of blood glucose is pivotal for efficient management of metabolic disorders such as diabetes, drastically reducing discomfort, costs, and waste associated with current invasive methods to measure blood glucose concentration.

The study will add to the body of evidence evaluating AI for use in the detection of glycaemic events (e.g., hypoglycaemia) in free-living conditions. Current efforts in the literature have been limited by small sample sizes[44], healthy subject populations[16], and data originating from highly controlled experimental settings[45].

Non-invasive monitoring of blood glucose and glycaemic event detection can potentially eliminate the need for finger-pricking. A continuous non-invasive blood glucose monitoring solution could help to overcome barriers and limitations of traditional methods, thereby increasing adherence to self-management protocols leading to improved outcomes, quality of life, and reduced incidence of complications.

Ethics and dissemination

This study has received ethical approval from the Research Ethics Service (Ref: 17/NW/0277). It is anticipated that the scientific findings of the study will be disseminated via presentation at national or international conferences and through publication in peer-reviewed scientific journals.

Author contributions

LP and JH conceived the idea. OC, KS, BO, MSH, JH, and LP contributed to the development of the protocol, study design and methods. OC wrote the first draft. KS, BO, MSH, TMB, MOW, JH, and LP critically revised the draft. All authors have approved the final written manuscript.

Funding statement

This study is funded by the Warwick-Wellcome Trust Translational Partnership Award and the Engineering and Physical Sciences Research Council Impact Acceleration Account (EPSRC IAA).

Competing interests statement

The authors have no competing interests to declare.

For peer review only

References

1. Lasker SP, McLachlan C, Wang L, et al. Discovery, treatment and management of diabetes. *Journal of Diabetology* 2010;Feb. 2010(1):1-8.
2. Atkinson MA, Eisenbarth GS, Michels AW. Type 1 diabetes. *The Lancet* 2014;383(9911):69-82. doi: https://doi.org/10.1016/S0140-6736(13)60591-7
3. McCall AL. Insulin therapy and hypoglycemia. *Endocrinol Metab Clin North Am* 2012;41(1):57-87. doi: 10.1016/j.ecl.2012.03.001 [published Online First: 2012/04/17]
4. Diabetes U. Us, diabetes and a lot of facts and stats. *London: Diabetes UK* 2019
5. Mobasseri M, Shirmohammadi M, Amiri T, et al. Prevalence and incidence of type 1 diabetes in the world: a systematic review and meta-analysis. *Health promotion perspectives* 2020;10(2):98.
6. White NH. Long-term outcomes in youths with diabetes mellitus. *Pediatric Clinics* 2015;62(4):889-909.
7. Nathan DM, Group DER. The diabetes control and complications trial/epidemiology of diabetes interventions and complications study at 30 years: overview. *Diabetes care* 2014;37(1):9-16.
8. Yoo HJ, An HG, Park SY, et al. Use of a real time continuous glucose monitoring system as a motivational device for poorly controlled type 2 diabetes. *Diabetes Research and Clinical Practice* 2008;82(1):73-79. doi: https://doi.org/10.1016/j.diabres.2008.06.015
9. Mogre V, Johnson NA, Tzelepis F, et al. A systematic review of adherence to diabetes self-care behaviours: Evidence from low- and middle-income countries. *Journal of Advanced Nursing* 2019;75(12):3374-89. doi: https://doi.org/10.1111/jan.14190
10. Nyomba BLG, Berard L, Murphy LJ. The Cost of Self-Monitoring of Blood Glucose Is an Important Factor Limiting Glycemic Control in Diabetic Patients. *Diabetes Care* 2002;25(7):1244-45. doi: 10.2337/diacare.25.7.1244-a
11. Ginsberg BH. Factors Affecting Blood Glucose Monitoring: Sources of Errors in Measurement. *Journal of Diabetes Science and Technology* 2009;3(4):903-13. doi: 10.1177/193229680900300438
12. Hypoglycemia ADAWo. Defining and Reporting Hypoglycemia in Diabetes: A report from the American Diabetes Association Workgroup on Hypoglycemia. *Diabetes Care* 2005;28(5):1245-49. doi: 10.2337/diacare.28.5.1245
13. Cryer PE, Davis SN, Shamoon H. Hypoglycemia in Diabetes. *Diabetes Care* 2003;26(6):1902-12. doi: 10.2337/diacare.26.6.1902
14. Parkin CG, Holloway M, Truesdell J, et al. Is Continuous Glucose Monitoring Underappreciated in the UK? *Eur Endocrinol* 2017;13(2):76-80. doi: 10.17925/EE.2017.13.02.76 [published Online First: 2017/08/22]
15. Porumb M, Pecchia L, Griffen C, et al. Nocturnal low glucose detection in healthy elderly from one-lead ECG using convolutional denoising autoencoders. *Biomedical Signal Processing and Control* 2020;62:102054. doi: http://dx.doi.org/10.1016/j.bspc.2020.102054
16. Porumb M, Pecchia L, Stranges S, et al. Precision Medicine and Artificial Intelligence: A Pilot Study on Deep Learning for Hypoglycemic Events Detection based on ECG. *Scientific reports* 2020;10(1):170. doi: http://dx.doi.org/10.1038/s41598-019-56927-5
17. Robinson RT, Harris ND, Ireland RH, et al. Changes in cardiac repolarization during clinical episodes of nocturnal hypoglycaemia in adults with Type 1 diabetes. *Diabetologia* 2004;47(2):312-5. doi: 10.1007/s00125-003-1292-4 [published Online First: 2004/01/09]
18. Laitinen T, Lyyra-Laitinen T, Huopio H, et al. Electrocardiographic alterations during hyperinsulinemic hypoglycemia in healthy subjects. *Ann Noninvasive Electrocardiol* 2008;13(2):97-105. doi: 10.1111/j.1542-474X.2008.00208.x
19. Nuryani N, Ling SSH, Nguyen HT. Electrocardiographic signals and swarm-based support vector machine for hypoglycemia detection. *Annals of biomedical engineering* 2012;40(4):934-45.

For peer review only

20. Tobore I, Liu Y, Kandwal A, et al. Non-invasive Monitoring of Three Glucose Ranges Based on ECG by Using DBSCAN-CNN. *IEEE Journal of Biomedical and Health Informatics* 2021;25(9):3340-50. doi: http://dx.doi.org/10.1109/JBHI.2021.3072628
21. Igbe T, Li J, Kandwal A, et al. An absolute magnitude deviation of hrv for the prediction of prediabetes with combined artificial neural network and regression tree methods. *Artificial Intelligence Review* 2021:No-Specified. doi: http://dx.doi.org/10.1007/s10462-021-10040-0
22. Cordeiro R, Karimian N, Park Y. Hyperglycemia Identification Using ECG in Deep Learning Era. *Sensors (Basel, Switzerland)* 2021;21(18) doi: https://dx.doi.org/10.3390/s21186263
23. Ling SH, San PP, Lam HK, et al. Hypoglycemia detection: multiple regression-based combinational neural logic approach. *SOFT COMPUTING* 2017;21(2):543-53. doi: 10.1007/s00500-015-1809-z
24. Diouri O, Cigler M, Vettoretti M, et al. Hypoglycaemia detection and prediction techniques: A systematic review on the latest developments. *Diabetes/Metabolism Research and Reviews* 2021
25. Griffen C, Duncan M, Hattersley J, et al. Effects of resistance exercise and whey protein supplementation on skeletal muscle strength, mass, physical function, and hormonal and inflammatory biomarkers in healthy active older men: a randomised, double-blind, placebo-controlled trial. *Experimental Gerontology* 2022;158:111651. doi: https://doi.org/10.1016/j.exger.2021.111651
26. Lalkhen AG, McCluskey A. Clinical tests: sensitivity and specificity. *Continuing Education in Anaesthesia Critical Care & Pain* 2008;8(6):221-23. doi: 10.1093/bjaceaccp/mkn041
27. Carney CE, Buysse DJ, Ancoli-Israel S, et al. The consensus sleep diary: standardizing prospective sleep self-monitoring. *Sleep* 2012;35(2):287-302. doi: 10.5665/sleep.1642 [published Online First: 2012/02/02]
28. Olafsdottir AF, Attvall S, Sandgren U, et al. A clinical trial of the accuracy and treatment experience of the flash glucose monitor FreeStyle Libre in adults with type 1 diabetes. *Diabetes technology & therapeutics* 2017;19(3):164-72.
29. Haleem MS, Castaldo R, Pagliara SM, et al. Time adaptive ECG driven cardiovascular disease detector. *Biomedical Signal Processing and Control* 2021;70:102968. doi: https://doi.org/10.1016/j.bspc.2021.102968
30. Shaffer F, Ginsberg JP. An Overview of Heart Rate Variability Metrics and Norms. *Frontiers in Public Health* 2017;5 doi: 10.3389/fpubh.2017.00258
31. Makowski D, Pham T, Lau ZJ, et al. NeuroKit2: A Python toolbox for neurophysiological signal processing. *Behavior research methods* 2021;53(4):1689-96.
32. Van Gent P, Farah H, Van Nes N, et al. HeartPy: A novel heart rate algorithm for the analysis of noisy signals. *Transportation research part F: traffic psychology and behaviour* 2019;66:368-78.
33. Mahrishi M, Hiran KK, Meena G, et al. Machine Learning and Deep Learning in Real-Time Applications: IGI global 2020.
34. {TensorFlow}: A System for {Large-Scale} Machine Learning. 12th USENIX symposium on operating systems design and implementation (OSDI 16); 2016.
35. Pedregosa F, Varoquaux G, Gramfort A, et al. Scikit-learn: Machine learning in Python. *the Journal of machine Learning research* 2011;12:2825-30.
36. McKinney W. pandas: a foundational Python library for data analysis and statistics. *Python for high performance and scientific computing* 2011;14(9):1-9.
37. Pickup JC, Sutton AJ. Severe hypoglycaemia and glycaemic control in Type 1 diabetes: meta-analysis of multiple daily insulin injections compared with continuous subcutaneous insulin infusion. *Diabetic Medicine* 2008;25(7):765-74. doi: https://doi.org/10.1111/j.1464-5491.2008.02486.x

For peer review only

38. Seaquist ER, Anderson J, Childs B, et al. Hypoglycemia and Diabetes: A Report of a Workgroup of the American Diabetes Association and The Endocrine Society. *Diabetes Care* 2013;36(5):1384-95. doi: 10.2337/dc12-2480
39. Association AD. 13. Diabetes Care in the Hospital. *Diabetes Care* 2015;39(Supplement_1):S99-S104. doi: 10.2337/dc16-S016
40. Moghissi ES, Korytkowski MT, DiNardo M, et al. American Association of Clinical Endocrinologists and American Diabetes Association consensus statement on inpatient glycemic control. *Diabetes Care* 2009;32(6):1119-31. doi: 10.2337/dc09-9029 [published Online First: 2009/05/12]
41. Georga EI, Fotiadis DI, Tigas SK. 2 - Pathophysiology and Management of Type 1 Diabetes. In: Georga EI, Fotiadis DI, Tigas SK, eds. Personalized Predictive Modeling in Type 1 Diabetes: Academic Press 2018:15-35.
42. Corwell B, Knight B, Olivieri L, et al. Current diagnosis and treatment of hyperglycemic emergencies. *Emergency Medicine Clinics* 2014;32(2):437-52.
43. Van Ness-Otunnu R, Hack JB. Hyperglycemic Crisis. *The Journal of Emergency Medicine* 2013;45(5):797-805. doi: https://doi.org/10.1016/j.jemermed.2013.03.040
44. Elvebakk O, Tronstad C, Birkeland KI, et al. A multiparameter model for non-invasive detection of hypoglycemia. *Physiological Measurement* 2019;40(8):085004. doi: 10.1088/1361-6579/ab3676
45. Cichosz SL, Frystyk J, Hejlesen OK, et al. A Novel Algorithm for Prediction and Detection of Hypoglycemia Based on Continuous Glucose Monitoring and Heart Rate Variability in Patients With Type 1 Diabetes. *Journal of Diabetes Science and Technology* 2014;8(4):731-37. doi: 10.1177/1932296814528838

For peer review only

Development of an artificial intelligence system to identify hypoglycaemia via ECG in adults with type 1 diabetes: protocol for data collection under controlled and free-living conditions

Journal:	*BMJ Open*
Manuscript ID	bmjopen-2022-067899.R1
Article Type:	Protocol
Date Submitted by the Author:	20-Feb-2023
Complete List of Authors:	Cisuelo, Owain; University of Warwick, School of Engineering<br>Stokes, Katherine; University of Warwick,<br>Oronti, Busola; University of Warwick, School of Engineering<br>Haleem, Salman; University of Warwick, Advanced Biomedical Signal Processing and Intelligent eHealth Lab<br>Barber, Thomas; University Hospitals Coventry and Warwickshire NHS Trust; University of Warwick<br>Weickert, Martin; University Hospitals Coventry & Warwickshire NHS Trust, ; University of Warwick Warwick Medical School, Division of Translational and Systems Medicine<br>Pecchia, Leandro ; University of Warwick, School of Engineering<br>Hattersley, John; University Hospitals Coventry & Warwickshire NHS Trust, Human Metabolism Research Unit; University of Warwick, School of Engineering
**Primary Subject Heading**:	Health informatics
Secondary Subject Heading:	Diabetes and endocrinology
Keywords:	Health informatics < BIOTECHNOLOGY & BIOINFORMATICS, General diabetes < DIABETES & ENDOCRINOLOGY, General endocrinology < DIABETES & ENDOCRINOLOGY

SCHOLARONE™ Manuscripts

Development of an artificial intelligence system to identify hypoglycaemia via ECG in adults with type 1 diabetes: protocol for data collection under controlled and free-living conditions

Owain Cisuelo[1*], Katy Stokes[1], Iyabosola Busola Oronti[1], Muhammad Salman Haleem[1], Thomas M Barber[2,3,4], Martin O Weickert[2,3,4], Leandro Pecchia[1], John Hattersley[1,4]

[1]School of Engineering, University of Warwick, Coventry, CV4 7AL, UK
[2]Medical School, University of Warwick, Coventry, CV4 7HL, UK
[3]Department of Endocrinology and Diabetes, University Hospitals Coventry & Warwickshire NHS Trust, Coventry, CV2 2DX, UK
[4]Human Metabolism Research Unit, University Hospitals Coventry & Warwickshire NHS Trust, Coventry, CV2 2DX, UK

*Corresponding author: Owain.Cisuelo@Warwick.ac.uk

For peer review only

Abstract

Introduction Hypoglycaemia is a harmful potential complication in people with Type 1 diabetes mellitus (T1DM) and can be exacerbated in patients receiving treatment, such as insulin therapies, by the very interventions aiming to achieve optimal blood glucose levels. Symptoms can vary greatly, including, but not limited to, trembling, palpitations, sweating, dry mouth, confusion, seizures, coma, brain damage or even death if untreated. A pilot study with healthy (euglycaemic) participants previously demonstrated that hypoglycaemia can be detected non-invasively with Artificial Intelligence (AI) using physiological signals obtained from wearable sensors. This protocol provides a methodological description of an observational study for obtaining physiological data from people with T1DM. The aim of this work is to further improve the previously developed AI model and validate its performance for glycaemic event detection in people with T1DM. Such a model could be suitable for integrating into a continuous, non-invasive, glucose monitoring system, contributing towards improving surveillance and management of blood glucose for people with diabetes.

Methods and analysis This observational study aims to recruit thirty T1DM patients from a diabetes outpatient clinic at the University Hospital Coventry and Warwickshire for a two-phase study. The first phase involves attending an inpatient protocol for up to thirty-six hours in a calorimetry room under controlled conditions, followed by a phase of free-living, for up to three days, in which participants will go about their normal daily activities unrestricted. Throughout the study the participants will wear wearable sensors to measure and record physiological signals (e.g., electrocardiogram and continuous glucose monitor). Data collected will be used to develop and validate an AI model using state-of-the-art deep-learning methods.

Ethics and dissemination This study has received ethical approval from National Research Ethics Service (Ref: 17/NW/0277). The findings will be disseminated via peer-reviewed journals and presented at scientific conferences.

Trial registration number NCT05461144

Keywords Artificial Intelligence (AI); Deep learning; Non-invasive glycaemic event detection; Hypoglycaemia detection; ECG signal processing; Type 1 diabetes mellitus (T1DM).

Article Summary

Strengths and limitations of this study

- This two-phase study will obtain data in both the controlled environment of a metabolic chamber and in free-living conditions, overcoming limitations of previous studies limited to controlled conditions.
- The second phase of the protocol has been designed with non-compliance in mind in order to determine how a model developed in controlled settings works with real-world data.

- This is a single centre observational study aiming to recruit from the local population without discrimination of characteristics such as sex, ethnicity or race which may limit the generalisability of the results.

Introduction

Type 1 diabetes mellitus (T1DM) is a chronic disorder caused by autoimmune damage to the insulin producing pancreatic beta cells, leading to elevated blood glucose concentration (hyperglycaemia) (1). T1DM requires exogenous insulin treatment (2), of which hypoglycaemia is a potential side effect (3). The prevalence of T1DM globally is 5.9 per 10,000 with the incidence of 15 per 100,000 people (4, 5). In UK, the prevalence is reported as 5th highest with 8.6% and incidence of T1DM is rising 5% every year (5). There is no cure for diabetes, therefore, effective management can be pivotal in reducing risk of adverse events as well as delay the onset of long-term complications (6, 7).

The self-monitoring of blood glucose (SMBG) can play a key role in effective management of diabetes. SMBG can empower patients to adhere to lifestyle interventions, such as changes to diet and exercise regimen, as they have access to immediate feedback on the effects (8). Active participation from the patient in their care has been shown to improve outcomes such as reducing the risk of diabetic complications (9). Current methods for glucose monitoring have several limitations. The most common method for SMBG is an invasive 'finger prick test' in which a small sample of blood is collected from the fingertip and analysed by a handheld device called a glucometer. This invasive procedure has several documented barriers preventing SMBG such as inconvenience, cost, and discomfort (10). Moreover, the devices and associated consumables are prone to error if not operated correctly (11), requiring repeat attempts and generating unnecessary waste. Hypoglycaemia typically occurs during sleep (12), and those receiving insulin-therapy are at increased risk due to a combination of factors such as impaired counterregulatory-hormone response and warning symptoms going unobserved (13). This presents an obvious limitation to the effectiveness of an invasive 'finger prick test' as a tool for SMBG. An alternative to 'finger pricking' are continuous glucose monitoring (CGM) devices which require a small cannula inserted in the top layer of skin to continuously measure glucose in the fluid between the cells. They can be described as minimally invasive as after the initial fitting they are routinely worn continuously for up to fourteen days. However, despite being shown to be a beneficial tool for SMBG, national clinical guidelines for the UK do not recommend routine use of CGMs for the management of T1DM due to insufficient evidence demonstrating their efficacy and cost-effectiveness (14).

It has been demonstrated, by this group (15, 16) and others (17-19), that glycaemic events can affect certain cardiac characteristics, which can be extracted from readings of the electrical activity of the heart, the electrocardiogram (ECG). With increasing availability of wearable devices for tracking physiological signals, studies have been undertaken to investigate prediction of blood glucose concentration or glycaemic event detection using artificial intelligence (AI), by combining data or features extracted from the ECG with blood glucose measurements. Such an approach would address the need for non-invasive continuous blood glucose monitoring. Studies have shown promising predictive performance based on data collected from healthy subjects (15, 16, 20-22), or adolescent T1DM patients (19, 23). In a review of techniques for detecting hypoglycaemia, Diouri et. al, note that the use of ECG-based techniques has only been investigated in small cohort trails, and that the success of such approaches relies on further, ideally larger, trials and validation in patients with diabetes and cardiac diseases (24).

This protocol expands on an initial pilot, results of which are published (15), which made opportune use of applicable secondary data collected for a separate study (25) on healthy elderly males. The aim of the pilot was to use the data to create a personalised AI model for the detection of nocturnal hypoglycaemia. The developed and validated deep-learning model achieved 90% for specificity (ability to identify true positives) and sensitivity (ability to identify true negatives) which are clinically relevant metrics in relation to diagnosis of disease (26). The aim of this protocol is to build on this initial work, by obtaining new data to validate the technique for people with diabetes and test its applicability beyond controlled conditions, to that of normal living conditions.

Study Objectives

For peer review only

The purpose of this observational study is to acquire continuous physiological data from adults with T1DM over a period of several days. Data obtained from wearable sensors and recorded in diaries to track lifestyle activities will be used to carry out our primary and secondary objectives.

Primary Objective

The primary objective of this study is to further develop and validate in a new population (T1DM) our previously developed AI approaches for non-invasive hypoglycaemia detection. We will extract and analyse ECG-derived features from the raw signal and determine their relationship with the glycaemic status. State-of-the-art algorithms will be used to create a mathematical mapping from the ECG-derived features to the glycaemic status.

Secondary Objective

To examine the impact of physical activity and diet on glycaemic events and incorporate relevant features into the model.

Methods and analysis

Study setting

This two-phase observational study will be conducted at the Human Metabolism Research Unit (HMRU) and in free-living conditions. During phase 1, the participants attend an inpatient protocol at University Hospital Coventry and Warwickshire (UHCW) in a calorimetry room for up to thirty-six hours. The calorimetry room is a controlled environment which allows for precise regulation of parameters such as ambient temperature, air pressure, humidity, and subject behaviour enabling us to obtain high quality baseline ECG and glucose data from the wearable sensors. In phase 2 the same sensors will be used to collect data under free-living conditions, for a period of up to three days, during which the participants are free to go about their normal daily activities without restriction. Participants will be asked to complete diaries for daily activity, food intake and sleep.

Inclusion and Exclusion Criteria

The study will be open to all adult individuals living independently with T1DM who are comfortable with a stay in a calorimetry room. The inclusion and exclusion criteria are summarised in table 1.

Table 1. Inclusion and Exclusion Criteria

Inclusion Criteria
• Aged 18 years or older<br>• Without acute illness or ongoing clinical investigation<br>• Participants with an ongoing medical condition will only be included after detailed consultation with clinical and dietetics members of the team
**Exclusion Criteria**
• Children (under 18 years)<br>• Any adult who lacks decisional capacity<br>• Claustrophobic and/or isolophobic patients, or needle phobia<br>• Individuals who have undertaken recent abnormal exercise, radiation exposure within the preceding 24 hours of entering the whole-body calorimeter and feeling unwell in any way.<br>• Any medical/endocrine problem that could affect energy expenditure (e.g., thyroid problems, Cushing's syndrome)<br>• Chronic inflammatory disorders like rheumatoid arthritis, or long-term use of steroids or other immunomodulators like cyclosporine, azathioprine.<br>• Beta blockers<br>• Currently actively losing weight<br>• Depression or any psychiatric illness

Enrolment procedure

For peer review only

Participants will be recruited from the Warwickshire Institute for the Study of Diabetes, Endocrinology, and Metabolism clinic at UHCW. We aim to recruit up to thirty adult T1DM patients who may enrol in either or both phases of the study. The process of identifying potential participants began in September 2022 and the first session took place in December 2022. Participant recruitment will be an ongoing process, expected to conclude September 2023. All participants will receive a participant information sheet and written informed consent will be obtained.

Sample size

As this is an observational study, an exact sample size calculation is not possible. A target of thirty participants will be enrolled, based on a pragmatic approach which ensures sufficient data is available for analysis. Full participation in the protocol will yield 108 hours of continuous data per participant. We aim to capture the daily blood glucose fluctuations known as glycaemic variability (27), a feature of impaired glycose metabolism. However, it is not possible to anticipate how many hypoglycaemic episodes will be recorded. ECG data, labelled with corresponding glucose concentration, will be analysed in excerpts of variable length from individual heart beats (cardiac cycles) to several minutes, resulting in a large sample size.

Study Protocol

Calorimetry room

The participant will stay in the calorimetry room for up to thirty-six hours, during which there will be set times for meals, light exercise, and rest. Meals will be provided with known total energy and macronutrient content. Water will be provided ad libitum. While in the calorimetry room, wearable sensors will record physiological data continuously.

Other activities will be performed during this time, specifically: a) up to 12 serial blood samples of 30ml will be obtained from a peripheral venous catheter inserted at the beginning of phase 1 to measure venous glucose and insulin concentration, b) finger-prick blood samples will be taken periodically for capillary glucose measurements, c) up to 12 saliva samples will be taken to analyse salivary concentration of cortisol and melatonin, d) all urine will be collected as voided for protein oxidation analysis, e) movement and activity will be assessed by motion sensors, f) simple questionnaires will be used to measure food intake and activity as well as subjective aspects such as appetite, satiety, and wellness, and g) daytime blood pressure will be monitored using an ambulatory blood pressure monitor (hourly readings).

Free-Living

While free-living, the participant will continue to wear the monitoring devices to record ECG and glucose concentration. If tolerated, participants will also be fitted with an ambulatory blood pressure monitor for the first 24-hours of the free-living phase. Instructions will be provided on how to affix and operate the devices. During this time, participants will also be required to keep brief diaries detailing physical activity, food intake and sleep. The first is a daily activity diary, to be completed at the end of each day, detailing: any device removal and replacement, physical activity, food intake, alcohol intake and caffeine intake. The second is a standardised sleep diary, the Consensus Sleep Diary-M (28) to be completed on waking in the morning.

Devices

Continuous Glucose Monitoring

Continuous glucose levels will be measured using a FreeStyle Libre 2 flash glucose monitoring system, which can be worn for up to fourteen days. Although the sensor is sampling continuously, the glucose concentration is reported in fifteen-minute intervals. The sensor can store data for a period of eight hours. As such, data will be extracted at regular intervals using a smartphone preconfigured with the companion application. The sensor is water-resistant and can be used while bathing, showering, swimming, or exercising, and is worn on the back of the upper arm. The device has been evaluated for accuracy and user experience and found to have been generally well received by T1DM patients who used it for ten to fourteen days (29).

ECG

For peer review only

Medtronic Zephyr BioPatch is a CE marked device, indicating that the product complies with EU safety, health, and environmental requirements, which operates across one lead within an ECG amplitude range between 0.25 and 15 mV, with a sampling frequency of 250Hz. The device will be affixed to the wearer's skin via two electrodes placed in the centre of the chest. It can also be worn in a secondary configuration attached to a fabric harness which is worn across the chest if the electrodes are not tolerated. The Zephyr BioPatch also has additional sensors to record physiological parameters such as activity levels, posture, and breathing rate which are all reported at 1Hz (30). The device will be removed before bathing, showering, or swimming and reaffixed afterwards. The battery is rated for thirty-five hours of continual use. Each participant will be provided with fully charged devices for the free-living phase of the study.

Data Management

All data collected is owned by the UHCW National Health Service (NHS) Trust. All electronic data, physiological or otherwise, generated as part of this study will be anonymised and stored in the HMRU database on secure servers and backed up and protected in accordance with NHS guidelines. Anonymised data will be made available to researchers for analysis under an Institutional Data Sharing Agreement between the UHCW and University of Warwick. Data will be extracted from the devices, anonymised, stored, and made available for analysis when the participant has concluded the protocol.

Data Analysis and Modelling

Data Processing

The effect of glycaemic events on the ECG signals will be examined. It is anticipated that ECG signals will be affected by noise and artefacts due to body movement or heavy respiration. Therefore, we will apply pre-processing methods such as baseline wander removal to the raw signal to remove low frequency noise. We will then identify individual heart beats in the ECG and detect fiducial points using our ECG segmentation tool (31) which has been used in a recent study for cardiovascular disease detection (32). Heartbeats will then be grouped into fifteen-minute excerpts, corresponding to the sampling of the CGM, and annotated with the glycaemic state (i.e., hypoglycaemic, normal, hyperglycaemic) according to the thresholds defined in Table 2. Due to the lower sample frequency of the CGM, we will perform linear interpolation to estimate glucose measurements at every second.

Table 2. Glycaemic event thresholds for people with T1DM (33-39).

Glycaemic event	Blood glucose concentration (mmol/L)
Severe hypoglycaemia	<2.8
Hypoglycaemia	>2.8 and <3.9
Euglycaemia	>3.9 and <11.1
Hyperglycaemia	>11.1 and <13.9
Severe hyperglycaemia	>13.9

Data Analysis

Data will be obtained from several days of continuous physiological monitoring of participants. During analysis the data will be divided into smaller excerpts such as 1, 2, or 5-minute intervals. In addition, beat-level ECG samples will be analysed, therefore the total number of samples available during analysis is ambiguous.

Characteristics of an ECG can be represented by heart rate variability (HRV) parameters and can be categorised into time-domain features, frequency-domain features and non-linear features (40). We aim to use physiological signal processing packages such as NeuroKit2 (41) and HeartPy (42) to determine these features. Furthermore, we aim to determine ECG beat parameters which include length and slope among the fiducial points (P, Q, R, S and T). We will perform statistical tests to determine the statistical significance of different HRV features and ECG morphology parameters among different glycaemic values to determine which parameters are associated with hypoglycaemia.

For peer review only

Covariates

The autonomic nervous system is responsible for maintaining homeostasis and regulates processes such as blood pressure (43), digestion (44), metabolism (45), and circadian rhythm (46), all of which may affect cardiac function and show as changes in the ECG morphology (47-50). Additionally, exercise can affect the QT interval, that is the ECG section representing ventricular depolarization, due to exercise-induced autonomic response (51). During phase 1 of the study daytime blood pressure will be monitored, and the calorific value and macronutrient composition of meals will be recorded. Additionally, exercise sessions will be scheduled throughout the phase. Throughout the free-living phase, all meals, exercise duration and intensity, and sleep times will be recorded by the participant. The data collected via this protocol enables us to examine how the covariates relate to the outcome of interest, i.e., the glycaemic status by performing statistical tests such as analysis of variance (ANOVA) (52). The recording of mealtimes, exercise, and sleep enables the data to be analysed during distinct periods of interest such as post-exercise or postprandial.

Data Modelling

The inherent part of the project is to develop AI models for detecting glycaemic events and inferring them with physical activity and diet. The state-of-the-art AI models vary from traditional machine learning models (such as Support Vector Machines, Decision Trees etc.) to advanced deep learning-based models (53). Traditional machine learning models have the capability to train static features for the development of explanatory models whereas deep learning models can train raw ECG signals based on spatial and temporal context. The deep learning models will be built upon earlier work developed for healthy subjects (15). We aim to develop and validate hypoglycaemia detection models that incorporate lifestyle trends such as activity levels and sleep patterns using metrics provided by the sensors and reported in the diaries. For a model to be clinically useful it should demonstrate generalisability. Therefore, we will test the model on unseen data using a subject-wise cross-validation approach (54).

Patient and public involvement

No formal PPI group was convened for this study, however, phase 1 is a standard protocol used at the HMRU and has been continually developed with PPI for over 10 years. The methodology using a single-lead wearable ECG device and CGM was discussed and reviewed with participants for a previous study investigating the effects of resistance exercise and protein supplementation on sarcopenia in healthy older men (clinical trials identifier: NCT03299972). The PPI consists of informal, non-scripted interviews with members of the public post-study via the UHCW PPI Forum. Depending on the results obtained, PPI groups may be approached to discuss non-scientific dissemination.

Discussion

The primary objective of this observational study is to obtain physiological data from people with T1DM using non-invasive wearable sensors to validate and further develop an AI model for automated detection of hypoglycaemia. The initial model (15) was developed using data from healthy individuals and the results determined that the personalised classifiers based on deep-learning algorithms can reliably perform automatic detection of hypoglycaemic events using features extracted from the ECG waveform recorded with wearable devices. This study is designed to improve the performance and robustness of this AI model for use in a population with T1DM. Additionally, as a secondary outcome, this study will examine impact of lifestyle habits such as exercise, diet, and sleep as predictors in the model.

The study will add to the body of evidence evaluating AI for use in the detection of glycaemic events (e.g., hypoglycaemia) in free-living conditions. Current efforts in the literature have been limited by small sample sizes (55), healthy subject populations (16), and data originating from highly controlled experimental settings (56). This study protocol has been developed to address the gaps of the existing literature. As such, the strengths of this study design are that data is collected from participants with diabetes, both in controlled and free-living settings. An additional strength of this protocol is that it has been designed with non-compliance in mind. The first phase will take place in a metabolic chamber where the activities of the participants and the operation of the wearable sensors are prescriptive and supervised. This enables us to obtain high quality baseline data. The second phase will take place in free-living conditions where the participants go about their normal daily activities unencumbered by the wearable sensors, providing real-life data. During the free-living phase a level of non-compliance and variance is desirable in order to determine how our analysis and model works with real-world data. Non-compliance with the

For peer review only

protocol will not exclude the participant from inclusion in the analysis. Any deviations from the protocol will be noted. Participation in both phases of the study is not a requirement, therefore we are likely to obtain different number of samples for each participant. We will take a pragmatic approach and work with the data available at end of the study. All analysis will report the number of samples used and if excerpts of data have been excluded and for what reason. Our planned analysis will segment the continuous physiological data into smaller excerpts of variable length, yielding a sufficient sample size for the training algorithm to discover relevant features in the data.

A potential limitation of this protocol is that during the free-living phase of the study participants will be required to extract data from the CGM at least once every eight hours. They will also have to remove and reattach the ECG device when engaging in water-based activities or exchanging the device for a new fully charged one. This introduces scenarios with the possibility of missing data such as if a CGM scan is missed, the ECG device is not changed when the battery is depleted, or it is not correctly activated. To mitigate this, participants will be provided with instructions and a demonstration as well as an information sheet. A member of the research team will also be contactable throughout if further assistance is needed.

To our knowledge no studies have attempted to develop a glycaemic event detection model for people with T1DM using deep-learning with raw single-lead ECG signals. This study is being run complementary and in parallel to another involving paediatrics with T1DM (57). In our planned analysis we will investigate changes in the ECG morphology and other features derived from the ECG signal with respect to glycaemic status. A novel aspect of the analysis will be the inclusion of additional features such as activity levels, mealtimes and composition, and sleep patterns.

Non-invasive monitoring of blood glucose and glycaemic event detection can potentially eliminate the need for finger-pricking. The development of an accurate and robust model for the non-invasive detection of abnormal excursions of blood glucose is pivotal for efficient management of metabolic disorders such as diabetes, drastically reducing discomfort, costs, and waste associated with current invasive methods to measure blood glucose concentration. A continuous non-invasive blood glucose monitoring solution could help to overcome barriers and limitations of traditional methods, thereby increasing adherence to self-management protocols leading to improved outcomes, quality of life, and reduced incidence of complications.

Ethics and dissemination

This study has received ethical approval from the Research Ethics Service (Ref: 17/NW/0277). It is anticipated that the scientific findings of the study will be disseminated via presentation at national or international conferences and through publication in peer-reviewed scientific journals.

Author contributions

LP and JH conceived the idea. OC, KS, BO, MSH, JH, and LP contributed to the development of the protocol, study design and methods. OC wrote the first draft. KS, BO, MSH, TMB, MOW, JH, and LP critically revised the draft. All authors have approved the final written manuscript.

Funding statement

This study is funded by the Warwick-Wellcome Trust Translational Partnership Award and the Engineering and Physical Sciences Research Council Impact Acceleration Account (EPSRC IAA).

Competing interests statement

The authors have no competing interests to declare.

References

1. Lasker SP, McLachlan C, Wang L, et al. Discovery, treatment and management of diabetes. Journal of Diabetology. 2010;Feb. 2010(1):1-8.

For peer review only

2. Atkinson MA, Eisenbarth GS, Michels AW. Type 1 diabetes. The Lancet. 2014;383(9911):69-82.
3. McCall AL. Insulin therapy and hypoglycemia. Endocrinol Metab Clin North Am. 2012;41(1):57-87.
4. Mobasseri M, Shirmohammadi M, Amiri T, et al. Prevalence and incidence of type 1 diabetes in the world: a systematic review and meta-analysis. Health promotion perspectives. 2020;10(2):98.
5. Diabetes UK. Us, diabetes and a lot of facts and stats.[online]. 2019. (accessed 23 January 2023). Available from: https://www.diabetes.org.uk/resources-s3/2019-11/facts-stats-update-oct-2019.pdf
6. White NH. Long-term outcomes in youths with diabetes mellitus. Pediatric Clinics. 2015;62(4):889-909.
7. Nathan DM, Group DER. The diabetes control and complications trial/epidemiology of diabetes interventions and complications study at 30 years: overview. Diabetes care. 2014;37(1):9-16.
8. Yoo HJ, An HG, Park SY, et al. Use of a real time continuous glucose monitoring system as a motivational device for poorly controlled type 2 diabetes. Diabetes Research and Clinical Practice. 2008;82(1):73-9.
9. Mogre V, Johnson NA, Tzelepis F, et al. A systematic review of adherence to diabetes self-care behaviours: Evidence from low- and middle-income countries. Journal of Advanced Nursing. 2019;75(12):3374-89.
10. Nyomba BLG, Berard L, Murphy LJ. The Cost of Self-Monitoring of Blood Glucose Is an Important Factor Limiting Glycemic Control in Diabetic Patients. Diabetes Care. 2002;25(7):1244-5.
11. Ginsberg BH. Factors Affecting Blood Glucose Monitoring: Sources of Errors in Measurement. Journal of Diabetes Science and Technology. 2009;3(4):903-13.
12. Hypoglycemia ADAWo. Defining and Reporting Hypoglycemia in Diabetes: A report from the American Diabetes Association Workgroup on Hypoglycemia. Diabetes Care. 2005;28(5):1245-9.
13. Cryer PE, Davis SN, Shamoon H. Hypoglycemia in Diabetes. Diabetes Care. 2003;26(6):1902-12.
14. Parkin CG, Holloway M, Truesdell J, et al. Is Continuous Glucose Monitoring Underappreciated in the UK? Eur Endocrinol. 2017;13(2):76-80.
15. Porumb M, Pecchia L, Griffen C, et al. Nocturnal low glucose detection in healthy elderly from one-lead ECG using convolutional denoising autoencoders. Biomedical Signal Processing and Control. 2020;62:102054.
16. Porumb M, Pecchia L, Stranges S, et al. Precision Medicine and Artificial Intelligence: A Pilot Study on Deep Learning for Hypoglycemic Events Detection based on ECG. Scientific reports. 2020;10(1):170.
17. Robinson RT, Harris ND, Ireland RH, et al. Changes in cardiac repolarization during clinical episodes of nocturnal hypoglycaemia in adults with Type 1 diabetes. Diabetologia. 2004;47(2):312-5.
18. Laitinen T, Lyyra-Laitinen T, Huopio H, et al. Electrocardiographic alterations during hyperinsulinemic hypoglycemia in healthy subjects. Ann Noninvasive Electrocardiol. 2008;13(2):97-105.
19. Nuryani N, Ling SSH, Nguyen HT. Electrocardiographic signals and swarm-based support vector machine for hypoglycemia detection. Annals of biomedical engineering. 2012;40(4):934-45.
20. Tobore I, Liu Y, Kandwal A, et al. Non-invasive Monitoring of Three Glucose Ranges Based on ECG by Using DBSCAN-CNN. IEEE Journal of Biomedical and Health Informatics. 2021;25(9):3340-50.
21. Igbe T, Li J, Kandwal A, et al. An absolute magnitude deviation of hrv for the prediction of prediabetes with combined artificial neural network and regression tree methods. Artificial Intelligence Review. 2021:No-Specified.
22. Cordeiro R, Karimian N, Park Y. Hyperglycemia Identification Using ECG in Deep Learning Era. Sensors (Basel, Switzerland). 2021;21(18).

For peer review only

23. Ling SH, San PP, Lam HK, et al. Hypoglycemia detection: multiple regression-based combinational neural logic approach. SOFT COMPUTING. 2017;21(2):543-53.
24. Diouri O, Cigler M, Vettoretti M, et al. Hypoglycaemia detection and prediction techniques: A systematic review on the latest developments. Diabetes/Metabolism Research and Reviews. 2021.
25. Griffen C, Duncan M, Hattersley J, et al. Effects of resistance exercise and whey protein supplementation on skeletal muscle strength, mass, physical function, and hormonal and inflammatory biomarkers in healthy active older men: a randomised, double-blind, placebo-controlled trial. Experimental Gerontology. 2022;158:111651.
26. Lalkhen AG, McCluskey A. Clinical tests: sensitivity and specificity. Continuing Education in Anaesthesia Critical Care & Pain. 2008;8(6):221-3.
27. Suh S, Kim JH. Glycemic Variability: How Do We Measure It and Why Is It Important? Diabetes Metab J. 2015;39(4):273-82.
28. Carney CE, Buysse DJ, Ancoli-Israel S, et al. The consensus sleep diary: standardizing prospective sleep self-monitoring. Sleep. 2012;35(2):287-302.
29. Olafsdottir AF, Attvall S, Sandgren U, et al. A clinical trial of the accuracy and treatment experience of the flash glucose monitor FreeStyle Libre in adults with type 1 diabetes. Diabetes technology & therapeutics. 2017;19(3):164-72.
30. Zephyr Technology. Bioharness 3 Log Data Descriptions [online]. 2016 (accessed 23 January 2023). Available from: https://www.zephyranywhere.com/media/download/bioharness-log-data-descriptions-07-apr-2016.pdf.
31. Haleem MS, Pecchia L. A Deep Learning Based ECG Segmentation Tool for Detection of ECG Beat Parameters. 2022 IEEE Symposium on Computers and Communications (ISCC). 2022:1-4.
32. Haleem MS, Castaldo R, Pagliara SM, et al. Time adaptive ECG driven cardiovascular disease detector. Biomedical Signal Processing and Control. 2021;70:102968.
33. Pickup JC, Sutton AJ. Severe hypoglycaemia and glycaemic control in Type 1 diabetes: meta-analysis of multiple daily insulin injections compared with continuous subcutaneous insulin infusion. Diabetic Medicine. 2008;25(7):765-74.
34. Seaquist ER, Anderson J, Childs B, et al. Hypoglycemia and Diabetes: A Report of a Workgroup of the American Diabetes Association and The Endocrine Society. Diabetes Care. 2013;36(5):1384-95.
35. Association AD. 13. Diabetes Care in the Hospital. Diabetes Care. 2015;39(Supplement_1):S99-S104.
36. Moghissi ES, Korytkowski MT, DiNardo M, et al. American Association of Clinical Endocrinologists and American Diabetes Association consensus statement on inpatient glycemic control. Diabetes Care. 2009;32(6):1119-31.
37. Georga EI, Fotiadis DI, Tigas SK. 2 - Pathophysiology and Management of Type 1 Diabetes. In: Georga EI, Fotiadis DI, Tigas SK, editors. Personalized Predictive Modeling in Type 1 Diabetes: Academic Press; 2018. p. 15-35.
38. Corwell B, Knight B, Olivieri L, et al. Current diagnosis and treatment of hyperglycemic emergencies. Emergency Medicine Clinics. 2014;32(2):437-52.
39. Van Ness-Otunnu R, Hack JB. Hyperglycemic Crisis. The Journal of Emergency Medicine. 2013;45(5):797-805.
40. Shaffer F, Ginsberg JP. An Overview of Heart Rate Variability Metrics and Norms. Frontiers in Public Health. 2017;5.
41. Makowski D, Pham T, Lau ZJ, et al. NeuroKit2: A Python toolbox for neurophysiological signal processing. Behavior research methods. 2021;53(4):1689-96.
42. Van Gent P, Farah H, Van Nes N, et al. HeartPy: A novel heart rate algorithm for the analysis of noisy signals. Transportation research part F: traffic psychology and behaviour. 2019;66:368-78.
43. Fernandez S, Srikakarlapudi S, Izzo JL. Chapter 73 - Blood Pressure Variability. In: Robertson D, Biaggioni I, Burnstock G, Low PA, Paton JFR, editors. Primer on the Autonomic Nervous System (Third Edition). San Diego: Academic Press; 2012. p. 355-7.

For peer review only

44. Camilleri M. Chapter 42 - Gastrointestinal Function. In: Robertson D, Biaggioni I, Burnstock G, Low PA, Paton JFR, editors. Primer on the Autonomic Nervous System (Third Edition). San Diego: Academic Press; 2012. p. 205-9.
45. Bell C. Chapter 52 - Regulation of Metabolism. In: Robertson D, Biaggioni I, Burnstock G, Low PA, Paton JFR, editors. Primer on the Autonomic Nervous System (Third Edition). San Diego: Academic Press; 2012. p. 253-5.
46. Golombek DA. Chapter 32 - Circadian Rhythms and Autonomic Function. In: Robertson D, Biaggioni I, Burnstock G, Low PA, Paton JFR, editors. Primer on the Autonomic Nervous System (Third Edition). San Diego: Academic Press; 2012. p. 157-9.
47. Widerlöv E, Jostell KG, Claesson L, et al. Influence of food intake on electrocardiograms of healthy male volunteers. European Journal of Clinical Pharmacology. 1999;55(9):619-24.
48. Hassing GJ, van der Wall HEC, van Westen GJP, et al. Blood pressure-related electrocardiographic findings in healthy young individuals. Blood Pressure. 2020;29(2):113-22.
49. Bonnemeier H, Wiegand UKH, Braasch W, et al. Circadian Profile of QT Interval and QT Interval Variability in 172 Healthy Volunteers. Pacing and Clinical Electrophysiology. 2003;26(1p2):377-82.
50. Dilaveris PE, Färbom P, Batchvarov V, et al. Circadian Behavior of P-Wave Duration, P-Wave Area, and PR Interval in Healthy Subjects. Annals of Noninvasive Electrocardiology. 2001;6(2):92-7.
51. Lecocq B, Lecocq V, Jaillon P. Physiologic relation between cardiac cycle and QT duration in healthy volunteers. The American Journal of Cardiology. 1989;64(8):481-6.
52. Introduction to analysis of variance. In: Davey AJH, Doncaster CP, editors. Analysis of Variance and Covariance: How to Choose and Construct Models for the Life Sciences. Cambridge: Cambridge University Press; 2007. p. 1-41.
53. Mahrishi M, Hiran KK, Meena G, et al. Machine Learning and Deep Learning in Real-Time Applications: IGI global; 2020.
54. Tougui I, Jilbab A, Mhamdi JE. Impact of the Choice of Cross-Validation Techniques on the Results of Machine Learning-Based Diagnostic Applications. Healthc Inform Res. 2021;27(3):189-99.
55. Elvebakk O, Tronstad C, Birkeland KI, et al. A multiparameter model for non-invasive detection of hypoglycemia. Physiological Measurement. 2019;40(8):085004.
56. Cichosz SL, Frystyk J, Hejlesen OK, et al. A Novel Algorithm for Prediction and Detection of Hypoglycemia Based on Continuous Glucose Monitoring and Heart Rate Variability in Patients With Type 1 Diabetes. Journal of Diabetes Science and Technology. 2014;8(4):731-7.
57. Andellini M, Haleem S, Angelini M, et al. Artificial intelligence for non-invasive glycaemic-events detection via ECG in a paediatric population: study protocol. Health and Technology. 2023;13(1):145-54.

For peer review only
